# ﻿New species and barcode analysis of *Bethylus* Latreille (Hymenoptera, Bethylidae) from South Korea

**DOI:** 10.3897/zookeys.1238.145564

**Published:** 2025-05-15

**Authors:** Kyung Min Lee, Il-kwon Kim, Jongok Lim

**Affiliations:** 1 Zoology Unit, Finnish Museum of Natural History, University of Helsinki, Helsinki, Finland; 2 Department of Forest Biodiversity, Korea National Arboretum, Pocheon, Gyeonggi Province, Republic of Korea; 3 Department of Life and Environmental Sciences, College of Agricultural and Food Sciences, Wonkwang University, Jeonbuk Province, Republic of Korea; 4 Institute of Life Science and Natural Resources, Wonkwang University, Jeonbuk Province, Republic of Korea

**Keywords:** Cytochrome c oxidase subunit I, ectoparasitoid, flat wasp, Korean Peninsula, taxonomy

## Abstract

For the first time, *Bethylus* Latreille, the type genus of Bethylidae (Hymenoptera, Chrysidoidea, Bethylinae), is reported from the Korean Peninsula based on the discovery of a new species, *Bethyluscolligatus***sp. nov.**, which was collected in Gangwon Province near the Demilitarized Zone. This new species is described and illustrated. A phylogenetic analysis was conducted using mitochondrial cytochrome c oxidase subunit I sequences from common *Bethylus* species to better understand the evolutionary relationships within the genus. The resulting phylogeny supports the distinction between *B.colligatus***sp. nov.** and other species. Additionally, a key to the species of *Bethylus* in East Asia is provided.

## ﻿Introduction

The ectoparasitoid family Bethylidae (Hymenoptera, Chrysidoidea) is found in all zoogeographic regions worldwide, but most of the species are concentrated in the tropics (Azevedo 1999; Azevedo et al. 2015, 2018). The family comprises approximately 131 genera and 3,314 described species, including 103 extinct species belonging to 28 genera across seven extant subfamilies Bethylinae, Chilepyrinae, Epyrinae, Glenoseminae, Mesitiinae, Pristocerinae, and Scleroderminae and five extinct subfamilies Cretabythinae, Elektroepyrinae, Holopsenellinae, and Protopristocerinae (Santos et al. 2024).

As of 2023, 48 species of Bethylidae had been recorded from the Korean Peninsula, with majority of the data coming from South Korea and no available records from North Korea. Recently, one *Pararhabdepyris* Gorbatovsky and four *Laelius* Ashmead species were described and added to the Korean flat wasp fauna (Lim and Kim 2024; Lim and Lee 2024), but only seven species belonging to two genera, *Goniozus* Förster and *Odontepyris* Kieffer, within Bethylinae have been reported in Korea (Lim et al. 2009; Lim and Lee 2012).

The type genus, *Bethylus* Latreille, 1802, is characterized by the following traits: second and third antennomeres equal in size, mandibles blunt and barely bidentate, head oval and depressed, compound eye small and non-prominent, mesosoma nearly uniformly wide or gradually tapering, and metasoma oval. The genus comprises 47 species primarily from the Palaearctic and Nearctic regions (Azevedo et al. 2018; Wang et al. 2021). In East Asia, 13 *Bethylus* species have been recorded: one from the Russian Far East (*B.fuscicornis* (Jurine)) (Gorbatovskij 1995), three from Japan (*B.fuscicornis* (Jurine), *B.sarobetsuensis* Terayama, and *B.shiganus* Terayama) (Terayama 2006), and 10 from China (including *B.sinensis* Xu, He & Terayama) (Xu et al. 2002; Wang et al. 2021). However, to date, no species of this genus from the Korean Peninsula have been reported.

Limited ecological information on *Bethylus* is available. Females of *B.cephalotes* Förster and *B.fuscicornis* sting and malaxate their relatively large prey and then drag it to a concealed location, such as a hollow stem. Several eggs are laid on the prey, and multiple larvae develop on a single host (Richards 1952). Additionally, some species parasitize olethreutid moths and nitidulid beetles in the United States (Evans 1962).

In this study, we report the first record of *Bethylus* from Korea, based on the newly described species *Bethyluscolligatus* sp. nov., which was collected in Gangwon Province near the Demilitarized Zone (DMZ). The identity of this new species is confirmed through molecular data, including mitochondrial cytochrome c oxidase subunit I (COI) sequences and phylogenetic analysis, which support its distinctiveness from other *Bethylus* species. We provide a detailed description and illustrations of the diagnostic characteristics of this new species, along with a key to the species of *Bethylus* in East Asia.

## ﻿Materials and methods

**Figure 1.**
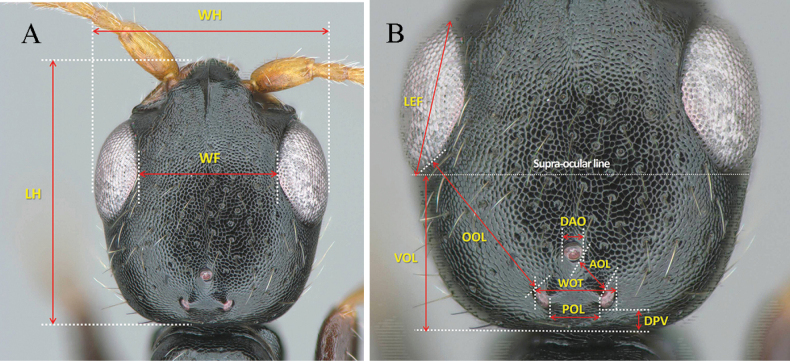
Biometric measurements for head habitus **A** overall measurements of head, dorsal view **B** measurements related with eye and ocelli, dorsal view. See Table 1 for abbreviations.

**Figure 2.**
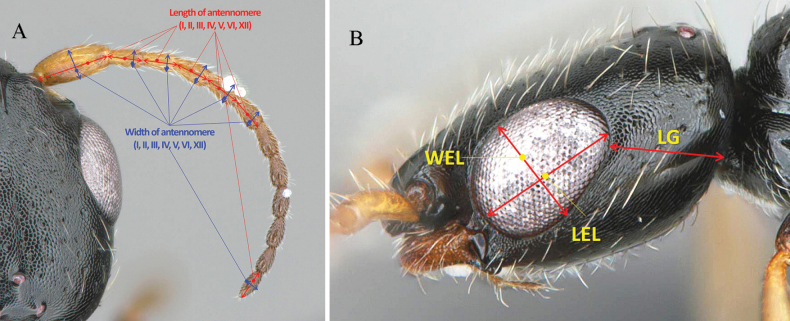
Biometric measurements for antennae and head habitus **A** measurements of antennomeres, dorsal view **B** measurements of head and gena, lateral view. See Table 1 for abbreviations.

**Figure 3.**
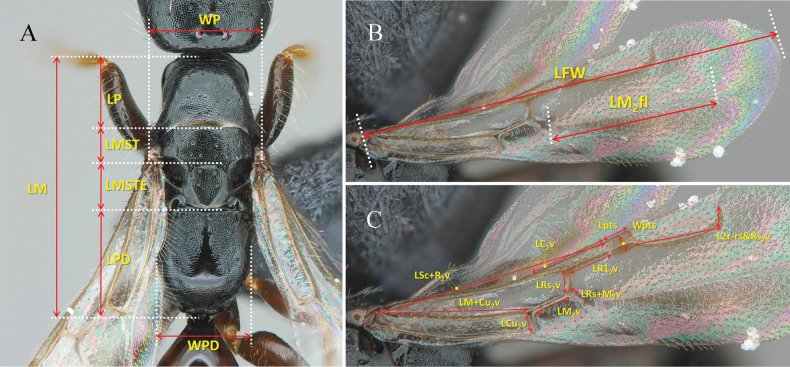
Biometric measurements for mesosoma and forewing **A** measurements of mesosoma, dorsal view **B, C** measurements related with veins and flexion line, dorsal view. See Table 1 for abbreviations.

All specimens in this study were collected using a Malaise trap deployed in a botanical garden located in Yanggu-gun, Gangwon Province, near the DMZ, the national border between South and North Korea. The abbreviations for the primary biometric measurements followed Lim (2011), with additional suggested and illustrated measurements for ease of understanding by students and researchers. The abbreviations and explanations are provided in Table 1 and Figs 1–3. The terminology for the integument sculpture followed that of Eady (1968) and Harris (1979). The general morphological terms were based on Azevedo et al. (2018) and Lanes et al. (2020), and the terms for mesopleural structures were adapted from Brito et al. (2021).

**Table 1.** A checklist of abbreviations and explanations for biometric measurements of *Bethylus*.

Abbreviation	Explanation	Remark
LH (Fig. 1A)	Length of head; maximum length from median clypeal lobe to posterior margin of head	Dorsal
WH (Fig. 1A)	Width of head; maximum width from outer margin of eye to opposite outer margin of eye	Dorsal
WF (Fig. 1A)	Width of frons; minimum width from inner margin of eye to opposite inner margin of eye	Dorsal
LEF (Fig. 1B)	Length of eye in frontal view; maximum length between anterior margin of eye and posterior margin of eye	Dorsal
VOL (Fig. 1B)	Length between supra-ocular line to posterior margin of head	Dorsal
OOL (Fig. 1B)	Minimum length between eye and posterior ocellus	Dorsal
DAO (Fig. 1B)	Diameter of anterior ocellus	Dorsal
AOL (Fig. 1B)	Minimum length between anterior and posterior ocelli	Dorsal
WOT (Fig. 1B)	Width of posterior ocelli; maximum width including lateral margin of posterior ocelli	Dorsal
POL (Fig. 1B)	Inner width between posterior ocelli; minimum width between inner margin of posterior ocelli	Dorsal
DPV (Fig. 1B)	Width between posterior margin of posterior ocellus and posterior margin of head	Dorsal
LAI–LAVI, LAXII (Fig. 2A)	Length of antennomeres from I to VI, and XII	Dorsal
WAI–WAVI, WAXII (Fig. 2A)	Width of antennomeres from I to VI, and XII	Dorsal
WEL (Fig. 2B)	Width of eye; maximum width of eye	Lateral
LG (Fig. 2B)	Length of gena; minimum length of gena	Lateral
LEL (Fig. 2B)	Length of eye; maximum length of eye	Lateral
LM (Fig. 3A)	Length of mesosoma; maximum length from anterior margin of pronotal disc to posterior margin of propodeal declivity	Dorsal
WP (Fig. 3A)	Width of pronotum; maximum width of pronotum	Dorsal
LP (Fig. 3A)	Length of pronotal dorsal area; maximum median length	Dorsal
LMST (Fig. 3A)	Length of mesoscutum; maximum median length	Dorsal
LMSTE (Fig. 3A)	Length of mesoscutellum; maximum median length	Dorsal
LPD (Fig. 3A)	Length of propodeum; maximum length from anterior margin of metapectal-propodeal disc to posterior margin of propodeal declivity	Dorsal
WPD (Fig. 3A)	Width of propodeum; maximum width of metapectal-propodeal disc	Dorsal
LFW (Fig. 3B)	Length of forewing; maximum length from base of forewing (excluding tegula) to apical margin of forewing	Dorsal
LM_2_fl (Fig. 3B)	Length of M2fl	Dorsal
LSc+R_2_v (Fig. 3B)	Length of Sc+R_2_v	Dorsal
LC_2_v (Fig. 3B)	Length of C_2_v	Dorsal
Lpts (Fig. 3B)	Length of pterostigma, maximum length of pterostigma	Dorsal
Wpts (Fig. 3B)	Width of pterostigma, maximum width of pterostigma	Dorsal
LM+Cu_2_v (Fig. 3B)	Length of M+Cu_2_v	Dorsal
LRs_2_v (Fig. 3B)	Length of Rs_2_v	Dorsal
LR1_2_v (Fig. 3B)	Length of R1_2_v	Dorsal
L2r-rs&Rs_2_v (Fig. 3B)	Length of 2r-rs&Rs_2_v	Dorsal
LCu_2_ (Fig. 3B)v	Length of Cu_2_v	Dorsal
LM_2_v (Fig. 3B)	Length of M_2_v	Dorsal
LRs+M_2_v	Length of Rs+M_2_v	Dorsal

Specimens were examined using a Leica M205 C stereomicroscope (Leica Microsystems, Solms, Germany), and images were captured using a Dhyana 400D camera (TUCSEN CMOS, Fujian, China), attached to the microscope. Multi-stacked images were generated using Delta Multifocus v. 24 (Delta, South Korea), and Helicon Focus v. 8.2.2 software (HeliconSoft, Kharkiv, Ukraine). The final images were edited using Adobe Photoshop 2025 (Adobe Systems Inc., San Jose, CA, USA).

For DNA extraction, the whole body of each specimen, excluding the head, was preserved using a non-destructive method, following the procedure outlined by Santos et al. (2018). Genomic DNA was extracted using the QIAamp DNA Micro Kit (Qiagen, Venlo, The Netherlands) according to the manufacturer’s protocol at the DNA laboratory of the Finnish Museum of Natural History (Luomus), Helsinki, Finland. The extracted DNA was stored at −20 °C at the Luomus facility and then used to amplify the 5' end of the mitochondrial COI gene (approximately 650 bp). Amplification was conducted using either C1-J-1718 (Simon et al. 1994) or HCO (Folmer et al. 1994) as the forward primer, and LCO (Folmer et al. 1994) as the reverse primer, which were modified to include T7 and T3 promoter universal tails, respectively.

PCR reactions were performed in a total volume of 12.5 μl, including 1–2 μl of extracted DNA, 3–4 μl of Milli-Q H2O, 6.25 μl of 2x MyTaq HS Red Mix (Bioline Co., London, UK), and 0.625 μl of each primer (10 mM). PCR conditions consisted of initial denaturation at 95 °C for 5 min, followed by 39 cycles of 30 s at 96 °C, 30 s at 50 °C, and 90 s at 72 °C, with a final extension step at 72 °C for 10 min. Amplicons were purified by adding 1–2 μl of ExoSAP-IT PCR product cleanup reagent (Thermo Fisher Scientific, Waltham, MA, USA) to 10 μl of the PCR products. The purified products were sent to the Institute for Molecular Medicine, Finland Genomics Unit, Helsinki, for Sanger sequencing. The electropherograms were edited using BioEdit v. 7 (Hall 2011) and aligned with other sequences using the MUSCLE algorithm in MEGA v. 11 (Tamura et al. 2021). Intra- and interspecific variations were calculated using the Kimura 2-parameter (K2P) model of nucleotide substitution in MEGA v. 11.

Phylogenetic analysis was performed using a dataset of 61 sequences (Appendix 1), including those published by Roslin et al. (2022). The recently recorded Chinese species of *Bethylus* (Wang et al. 2021) were not included in this analysis because of the absence of sequence data. Four species of *Goniozus*, *G.claripennis* (Förster), *G.jacintae* Farrugia, *G.nephantidis* (Muesebeck), and *G.omanensis* Polaszek, were used as the outgroup. The GenBank accession numbers for the four newly generated *Bethylus* sequences from Korea are PQ777353–PQ777356. Phylogenetic trees were constructed using the maximum-likelihood (ML) method in IQ-TREE (Minh et al. 2020), and the best-fitting substitution models were selected using ModelFinder (Kalyaanamoorthy et al. 2017). Node support was assessed using ultrafast bootstrap approximations (UFBoot2) and an SH-like approximate likelihood ratio test (Hoang et al. 2018). To reduce overestimation of branch supports, the “-bnni” option was applied to optimize bootstrap trees using hill-climbing nearest-neighbor-interchange searches. The final phylogenetic tree was visualized in FigTree v. 1.4.2 (Rambaut 2015) and further edited using Adobe Illustrator CS6.

The specimens examined in this study were deposited in the Entomological Collection of the Korea National Arboretum (**KNAE**) in Pocheon, South Korea.

## ﻿Results

### ﻿Genus Bethylus

Taxon classificationAnimaliaHymenopteraBethylidae

Latreille, 1802

3C2F47CD-A8A1-5421-B733-6487C6874CAD

Bethylus Latreille, 1802: 315. Type species: Omalusfuscicornis Jurine, 1807.
Anoxus Thomson, 1862: 451. Type species: Anoxusboops Thomson, 1862.
Anoxys Dalla Torre, 1898: 550. Unjustified emendation to Anoxus by Dalla Torre (1898).
Perisemus Förster, 1856: 95. Type species: Bethylustriareolatus Förster, 1856.
Episemus Thomson, 1862: 452. Type species: Epysemusvariabilis Thomson, 1862 [1861].
Digoniozus Kieffer, 1905: 245. Type species: Perisemusoregonensis Ashmead, 1893.

#### Diagnosis.

Palpal formula 5:2; clypeus short and not strongly angulated medially; antenna with 12 antennomeres; notauli absent parapsidal signum present; anteromesoscutum short; metapectal-propodeal complex with lateral carina present, metapostnotal median carina absent; prosternum large, diamond-shaped; hypopygium with posterior bilobate; parameres double, completely divided into dorsal and ventral arms (Azevedo et al. 2018).

### ﻿ Bethylus colligatus

Taxon classificationAnimaliaHymenopteraBethylidae

Lim sp. nov.

EBFD6995-8B99-5DB2-9972-D2DF1FAE9D29

https://zoobank.org/D85910D7-3041-403C-83A8-F746A642F281

Figs 1 , 2 , 3 , 4

#### Description.

Holotype (female). ***Colour***. *Head*: black, mandible castaneous; antenna light castaneous, darkening apicad. *Mesosoma*: black; forewing hyaline and subhyaline, veins light castaneous; coxae, trochanters, femora dark castaneous; tibiae and tarsomere V light castaneous; tarsomeres I–V yellow. *Metasoma*: dark castaneous.

***Morphology*.** Body length 3.7 mm.

**Figure 4.**
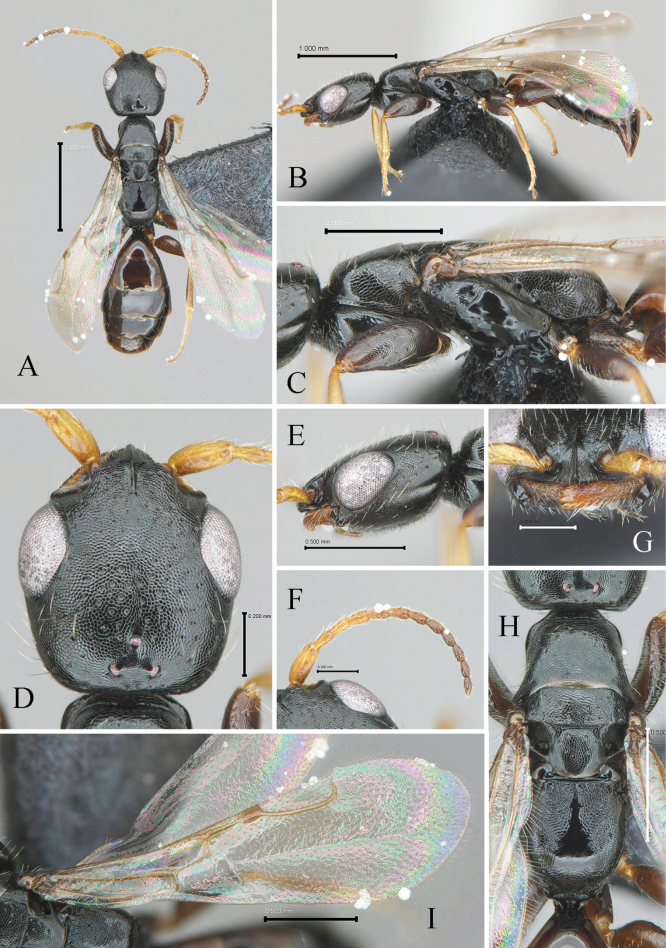
Diagnostic characteristics of *Bethyluscolligatus* sp. nov., female, holotype **A** whole body, dorsal view **B** whole body, lateral view **C** mesosoma, lateral view **D** head, dorsal view **E** head, lateral view **F** antennae, dorsal view **G** mandible, frontal view **H** mesosoma, dorsal view **I** forewing in dorsal view. Scale bars: 1 mm (**A, B**); 0.50 mm (**C, E, H, I**); 0.20 mm (**D–G**).

***Head*** (Fig. 4D–G): 1.1× as long as wide (Fig. 4D). Lateral margin posterior to eye tapering to postero-lateral corner, postero-lateral corner forming round angle, posterior margin straight in dorsal view with long sub-erect setae, vertex crest slightly outcurved (Fig. 4D). Mandible with hairs and four teeth; all teeth sharpened apically, getting long to ventralmost tooth (Fig. 4G). Clypeus well developed, median clypeal lobe distinctly protuberant with apex rounded; lateral clypeal lobe polished and smooth; median clypeal carina developed, slightly extending posterad into frons (Fig. 4D). Antenna with 12 antennomeres; each antennomere longer than wide; first six antennomeres with in ratio of 2.19:1.08:1.00:1.04:1.04:1.02 and 1.03 in length; 2.3, 1.9, 2.0, 1.8, 1.8, 1.8 and 2.2× as long as wide, respectively (Fig. 4F). Frons coriaceous with sparse shallow punctures, separated each other by 1.5–4.0× of their diameter (Fig. 4D). WF 1.2× LEF (Fig. 4D). Mediooccipito-genal suture present. Occipital carina absent. Compound eye 0.35 mm in dorsal view (Fig. 4D); 0.34 mm long and 1.4× as long a wide in lateral view (Fig. 4E); LEF 1.2× VOL (Fig. 4D). Anterior angle of ocellar triangle acute, POL1.2× AOL; OOL 2.0 × WOT; DPV 1.7 × DAO (Fig. 4D). LEL 1.3 × LG (Fig. 4E). Malar space narrow; malar line between mandible and eye absent. Gena coriaceous with shallow sparse punctures (Fig. 4E).

***Mesosoma*** (Fig. 4A–C, H): pronotal dorsal area coriaceous, 0.5× long as wide with sparse small punctures as dorsal surface of head; posterior margin outcurved medially (Fig. 4H). Mesoscutum coriaceous with few punctures; notaulus absent; parapsidal signum parallel, well developed, and continued to posterior carina between mesoscutum and mesoscutellum; posterior margin of mesoscutum broadly rounded (Fig. 4H). LMS 0.8× as long as LMST (Fig. 4H). Mesoscutellar disc coriaceous; mesoscuto-scutellar fovea elongate, oblique (Fig. 4H). Metanotum reduced medially; metanotal fovea present; lateral area polished and smooth (Fig. 4H). Metapectal-propodeal complex 1.1× as long as wide; metapostnotum depressed and reticulate; median ridge smooth and polished; metapectal-propodeal disc rugulose, depressed and obliquely rugose near lateral marginal carina of metapectal-propodeal disc; lateral marginal carina of metapectal-propodeal disc complete; propodeal declivity coriaceous; anterior metapleural area coriaceous, metapleural line with two distinct pits and some weak pits; lateral surface of metapectal-propodeal complex strongly coriaceous (Fig. 4H). Propleuron coriaceous. Mesopectus coriaceous with shallow punctures; subalar impression, mesepimeral sulcus distinct. Prosternum coriaceous with weak longitudinal sulcus medially.

***Forewing*** (Fig. 4I): 2.39 mm long. Rs+M_2_v extremely short. 2r-rs&Rs_2_v with apex angled curved. R1_2_v 1.4× as long as Lpts. M_2_v 2.0× as long as Rs_2_v. Rs_2_v 1.0× as long as Cu_2_v. R_2_c, 1Cu_2_c, and 1M_2_c hyaline with sparse, short hairs; elsewhere with denser, short hairs. M_2_fl 0.94 mm long, straight.

***Metasoma*** (Fig. 4A): metasomal terga smooth and polished, weakly coriaceous basally; longitudinal sulcus of first metasomal tergum distinctly exceeding first metasomal spiracle. Overlapping region of tergum weakly coriaceous. S3–S6 with one transverse apical line of small punctures with suberect hairs along with posterior margin of each sternite.

#### Type locality.

South Korea • Gangwon Province: Yanggu: Haean: Mandae: DMZ Botanical Garden; Malaise trap, 38°15'13"N, 128°6'47"E, Alt. 608 m, 30.vi.2015, H.T. Shin leg.

#### Type material.

***Holotype***: ♀, South Korea: Gangwon Province: Yanggu: Haean: Mandae: DMZ Botanical Garden; Malaise trap, 38°15'13"N, 128°6'47"E, Alt. 608 m, 30.vi.2015, H.T. Shin leg. (KNAE20150630-MT-055). Paratypes: 2 ♀, same data as holotype, KNAE20150630-MT-056, KNAE20150630-MT-057.

#### DNA barcodes.

GenBank accession numbers PQ777353–PQ777356. The intraspecific divergence of the barcode region averages 1.15%, with a maximum distance of 2.11% (*n* = 4). The minimum distance to its nearest neighbour, *B.berlandi*, is 22.0%.

#### Distribution.

Known only from the type locality.

#### Etymology.

The name *colligatus* is Latin, meaning “unified,” as the type specimens were collected from the DMZ area (Yanggu County, Gangwon Province), situated between the two Koreas. The species epithet is treated as an adjective in the nominative.

#### Remarks.

This species closely resembles *B.convexus* Wang, He & Chen from China in terms of body colour, the median clypeal lobe with a rounded apex, an acute anterior angle of the ocellar triangle, a distinctly protuberant eye, and being macropterous. However, it differs from *B.convexus* in several key features: the side of the head posterior to the eye taper towards the postero-lateral corner, all mandibular apical teeth are sharpened, the DPV is 1.7× the DAO, the apex of 2r-rs&Rs2v is distinctly angled in *B.colligatus* sp. nov., whereas the sides of head posterior to eye parallel, upper most one with margin truncated, the DPV is 1.0× the DAO, and the apex of 2r-rs&Rs2v is rounded in *B.convexus*.

#### Molecular data of *Bethylus*.

The final alignment of the mitochondrial COI sequences consists of 61 sequences with 707 bp, and 7.6% of the dataset contains gaps or missing data. Among the aligned sites, 289 are parsimony-informative, 42 are singleton, and 376 are constant. Nearest-neighbour distance analysis of *Bethylus* using the K2P model reveals a minimum interspecific distance of 16.6% between *B.fuscicornis* and *B.berlandi* and a maximum of 33.2% between *B.fuscicornis*/*B.boops* (Thomson) and *B.colligatus* (Table 2). In contrast, the maximum intraspecific distance is 2.1% within *B.colligatus* and 20.1% within *B.fuscicornis*, indicating deep genetic variation within the latter species.

**Table 2.** K2P intra- and interspecific distance (%) showing the range between minimum and maximum values among species of *Bethylus*.

Division	*B.fuscicornis*	*B.boops*	*B.cephalotes*	*B.berlandi*	*B.colligatus* sp. nov.
***B.fuscicornis***	0.0–20.1				
***B.boops***	19.3–24.9	0.0			
***B.cephalotes***	17.4–22.5	20.8	0.0		
***B.berlandi***	16.6–20.4	19.6	17.5	0.0	
***B.colligatus* sp. nov.**	27.9–33.2	32.7–33.2	31.4–31.8	22.0–22.4	0.0–2.1

**Figure 5.**
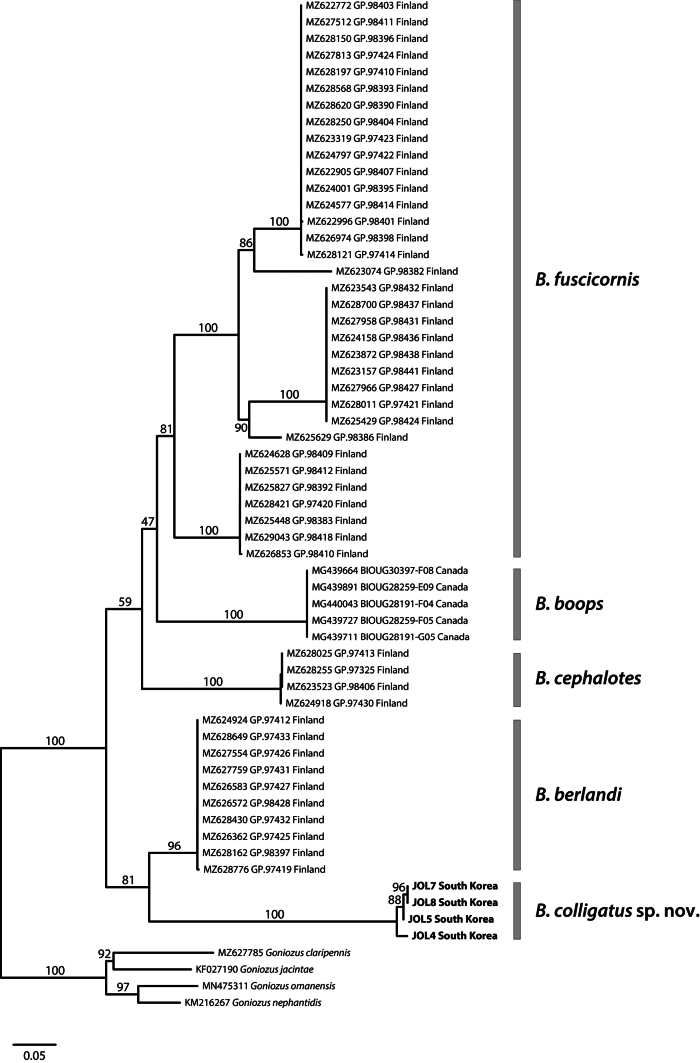
Maximum-likelihood (ML) tree based on COI sequences of *Bethylus* species, rooted with four *Goniozus* species as the outgroup. Numbers near the branches represent ultrafast bootstrap support values from the ML analysis.

Using the Bayesian Information Criterion, the best-fit substitution model for the dataset is GTR+F+I+G4. This model was subsequently applied to ML inference. Phylogenetic analysis of the mitochondrial COI dataset provided robust support for relationships among lineages and species (Fig. 5). The ML tree fully supports *B.colligatus* sp. nov., which is grouped as a sister taxon to *B.berlandi* with 81% bootstrap support. The Finnish *B.fuscicornis* is shown to have diverged into five distinct lineages, clustering with the Canadian *B.boops*. *Bethyluscephalotes* is sister to the clade comprising *B.fuscicornis* and *B.boops*.

### ﻿Key to *Bethylus* species from East Asia and distribution (modified from Wang et al. 2021; Terayama 2006)

**Table:** 

1	Mandible with three apical teeth	***Bethylussarobetsuensis* Terayama, 2006 (Japan)**
–	Mandible with more than four apical teeth	**2**
2	Mandible with four apical teeth	**3**
–	Mandible with five apical teeth	***Bethylusfuscicornis* (Jurine, 1807) (Russia, Japan)**
3	Median clypeal lobe broadly rounded	**4**
–	Median clypeal lobe truncated medially	**10**
4	POL less than 1.1× as wide as OL	**5**
–	POL more than 1.2× as wide as AOL	**7**
5	LH less than 1.1× as long as WH	**6**
–	LH more than 1.1× as long as WH	***Bethylusincurvus* Wang, He & Chen, 2021 (China)**
6	Distance of posterior ocellus to vertex crest more than 2.8 as wide as DAO	***Bethyluscrassicaptis* Wang, He & Chen, 2021 (China)**
–	Distance of posterior ocellus to vertex crest less than 1.5× as wide as DAO	***Bethylusglabricarinatus* Wang, He & Chen, 2021 (China)**
7	Ocelli large, DPV less than 0.5× as wide DAO	***Bethylusgansensis* Wang, He & Chen, 2021 (China)**
–	Ocelli small, DPV more than 0.5× as wide DAO	**8**
8	Eye not protuberant; median clypeal lobe short; brachypterous	***Bethylussinensis* Xu, He & Terayama, 2002 (China)**
–	Eye distinctly protuberant; median clypeal lobe long; macropterous	**9**
9	Side of head posterior to eye parallel; apex of 2r-rs&Rs_2_v with round angle	***Bethylusconvexus* Wang, He and Chen, 2021 (China)**
–	Side of head posterior to eye tapering; apex of 2r-rs&Rs_2_v with blunt angle	***Bethyluscolligatus* sp. nov. (Korea)**
10	POL less than 1.3× as wide AOL	**11**
–	POL less more 1.3× as wide AOL	**13**
11	DPV less than 1.5× as wide as DAO	***Bethylusprolatus* Wang, He & Chen, 2021 (China)**
–	DPV more than 2.0× as wide as DAO	**12**
12	Antennomere III less than 2.0× as long as wide; male	***Bethylusquadraticapitis* Wang, He & Chen, 2021 (China)**
–	Antennomere III more than 2.0× as long as wide; female	***Bethylusshiganus* Terayama, 2006 (Japan)**
13	Apex of cuspis narrow	***Bethylushunanensis* Wang, He & Chen, 2021 (China)**
–	Apex of cuspis rounded	***Bethylusningxicus* Wang, He & Chen, 2021 (China)**

## ﻿Discussion

Evans (1962) noted that the highly evolved *Bethylus* has a circumpolar distribution, with all described species found in the Holarctic region (Palaearctic and Nearctic regions) (Gordh and Móczár 1990; Azevedo et al. 2018; Wang et al. 2021). The unique Oriental species *B.amplipennis* (Motschulsky) was synonymized with *Holepyris* Kieffer by Krombein (1987). Only four *Bethylus* species have been recorded in Eastern Asia: *B.fuscicornis* found throughout Russia and Japan, *B.sarobetsuensis* and *B.shiganus* both from Japan, and *B.sinensis* from China. Recently, nine additional species were added to China fauna (Wang et al. 2021). To date, no *Bethylus* species from the Korean Peninsula have been recorded, but several specimens of a new species have been discovered in Gangwon Province near the DMZ in South Korea.

Body size is a significant morphological characteristic that is associated with various physiological and ecological traits and is influenced by both biotic and abiotic factors (McDonald and Ward 2023). Most taxonomic studies on Bethylidae provide biometric data on body size for each species. However, body size is highly dependent on the condition of the dried specimens and the preservation of the metasomal segments. To address this, Evans (1962) suggested using the ratio of forewing length to metatibia length, because it is easier to accurately measure than the total body length. Additionally, the cephalic region has numerous useful taxonomic and diagnostic characteristics, and some studies on Bethylidae have detailed how to measure these characteristics (Terayama 2006; Lim 2011; Brazidec et al. 2024). Given that many morphological terms and characteristics of Bethylidae have been recently revised (Azevedo et al. 2018; Lanes et al. 2020; Brito et al. 2021), we propose standard methods of measurement, including abbreviations, explanations, and illustrations, particularly for the head and forewings (Figs 1–3), to create a uniform system for students and researchers working with bethylids.

Although some genera of Bethylinae, such *Prosierola* Kieffer, *Lytopsenella* Kieffer, and *Afrobethylus* Ramos & Azevedo have no polymorphisms of forewing (Azevedo 2008, 2009; Ramos and Azevedo 2016), both sexes of most species of *Bethylus* and *Eupsenella* Westwood (Bethylinae) and *Acephalonomia* Strejček, *Bethylopsis* Fouts, *Cephalonomia* Westwood, *Glenosema* Kieffer, *Megaprostenum* Azevedo, *Platepyris* Lanes & Azevedo, and *Sclerodermus* Latreille (Scleroderminae) have different types of forewings: full-winged, brachypterous, and micropterous (Azevedo et al. 2018; Ramos and Azevedo 2012; Vargas et al. 2020). In *Bethylus*, especially the type species, *B.fuscicornis* has both full-winged and micropterous forms, whereas other species, such as *B.decipiens* (Provancher) and *B.amoenus* Fouts from Canada and the USA, have fully winged and brachypterous forms. *Bethylussinensis* from China has micropterous wings, whereas *B.sarobetsuensis* from Japan has brachypterous form. Most of the remaining species, including *B.colligatus* sp. nov., have full-winged forms. Although Bethylidae were not included in the analyses, Danforth (1989) mentioned that similar wing morphologies among distantly related species could result from similarities in body size, which has important implications in hymenopteran phylogeny, especially at lower taxonomic levels where wing characters are heavily utilized. Wing venation has been analyzed in evolutionary studies of Hymenoptera (Sharkey 2007; Perfilieva 2010; Ramos and Azevedo 2020; Anand et al. 2023). Evans (1962) emphasized forewing length in Bethylidae, whereas Azevedo et al. (2018) noted that wing length is polymorphic within species. Therefore, analysis of forewing characteristics is essential for understanding the evolutionary relationships among *Bethylus* species.

Although *Bethylus* is the type genus of Bethylidae, it is one of the smallest genera in the family and in Bethylinae, and this limits the number of species that can be included for morphological and molecular analyses. For example, no *Bethylus* species were included in the analysis of mesopleural structures in Bethylidae (Brito et al. 2021). Only four species have been included in phylogenomic analyses (Santos et al. 2024), and only a few species have been studied morphologically and molecularly (Magnacca 2024). Ramos and Azevedo (2020) used seven species in a morphological phylogenetic analysis of Bethylinae, in which *Bethylus* formed a clade with *Afrobethylus*, which was a sister to a clade containing *Sierola* Cameron. *Bethyluscolligatus* sp. nov. is the 48^th^ species described globally, but the number of species is still insufficient to fully understand the evolutionary interrelationships within the genus.

In this study, we examined the molecular relationships among four *Bethylus* species, including the newly described *B.colligatus*. Our results indicated that the new species is genetically distinct from its congeners, with particularly high genetic distances. The DNA barcodes for *Bethylus* diverged from 16.6% to 33.2% between species (Table 2), far exceeding the widely accepted 2% threshold for DNA barcoding (Hebert et al. 2003). This clear separation suggests that *B.colligatus* has evolved distinct genetic characteristics, possibly because of its long-term geographical isolation in the unique ecosystem of the Korean Peninsula, with limited gene flow from neighbouring species found in China and Japan.

In addition, we did not include the Chinese *Bethylus* species (Wang et al. 2021) and *B.fuscicornis* from the Russian Far East and Japan in our analysis because of the absence of COI sequence data. However, if we can collect *B.fuscicornis* in South Korea, we could study the evolutionary relationships between European and East Asian specimens.

Our findings raise questions about the applicability of the 2% threshold in *Bethylus* and possibly in Hymenoptera. For instance, a study on German cuckoo wasps reported high barcode variation, with divergences of up to 13% in *Holopygagenerosa* (Förster), which indicates the presence of cryptic species (Schmid-Egger et al. 2024). Similarly, research on the genus *Tanytarsus* van der Wulp (Diptera) revealed deep interspecific divergence, suggesting a 4–5% threshold (Lin et al. 2015). The unusually high divergence detected in *Bethylus* suggests that a 2% threshold may not be universally suitable for delimiting species in this genus. Consequently, an integrative approach that incorporates morphological, ecological, and genomic data is crucial for refining the species boundaries and advancing our understanding of the true diversity within *Bethylus*.

## Supplementary Material

XML Treatment for Bethylus

XML Treatment for Bethylus colligatus

## ﻿Additional information

### Author ORCIDs

Kyung Min Lee https://orcid.org/0000-0001-9055-1073

Il-kwon Kim https://orcid.org/0000-0002-3805-6590

Jongok Lim https://orcid.org/0000-0001-5700-3024

### Data availability

All of the data that support the findings of this study are available in the main text.

## ﻿Appendix 1

**Table A1.** A list of accession number for the species analyzed in the present study.

	GenBank	Sample ID	Species	Country
1	MZ624924	GP.97412	*Bethylusberlandi*	Finland
2	MZ628776	GP.97419	*Bethylusberlandi*	Finland
3	MZ626362	GP.97425	*Bethylusberlandi*	Finland
4	MZ627554	GP.97426	*Bethylusberlandi*	Finland
5	MZ626583	GP.97427	*Bethylusberlandi*	Finland
6	MZ627759	GP.97431	*Bethylusberlandi*	Finland
7	MZ628430	GP.97432	*Bethylusberlandi*	Finland
8	MZ628649	GP.97433	*Bethylusberlandi*	Finland
9	MZ628162	GP.98397	*Bethylusberlandi*	Finland
10	MZ626572	GP.98428	*Bethylusberlandi*	Finland
11	MG440043	BIOUG28191-F04	*Bethylusboops*	Canada
12	MG439711	BIOUG28191-G05	*Bethylusboops*	Canada
13	MG439891	BIOUG28259-E09	*Bethylusboops*	Canada
14	MG439727	BIOUG28259-F05	*Bethylusboops*	Canada
15	MG439664	BIOUG30397-F08	*Bethylusboops*	Canada
16	MZ628255	GP.97325	*Bethyluscephalotes*	Finland
17	MZ628025	GP.97413	*Bethyluscephalotes*	Finland
18	MZ624918	GP.97430	*Bethyluscephalotes*	Finland
19	MZ623523	GP.98406	*Bethyluscephalotes*	Finland
20	PQ777353	JOL4	*Bethyluscolligatus*	South Korea
21	PQ777354	JOL5	*Bethyluscolligatus*	South Korea
22	PQ777355	JOL7	*Bethyluscolligatus*	South Korea
23	PQ777356	JOL8	*Bethyluscolligatus*	South Korea
24	MZ628197	GP.97410	*Bethylusfuscicornis*	Finland
25	MZ628121	GP.97414	*Bethylusfuscicornis*	Finland
26	MZ628421	GP.97420	*Bethylusfuscicornis*	Finland
27	MZ628011	GP.97421	*Bethylusfuscicornis*	Finland
28	MZ624797	GP.97422	*Bethylusfuscicornis*	Finland
29	MZ623319	GP.97423	*Bethylusfuscicornis*	Finland
30	MZ627813	GP.97424	*Bethylusfuscicornis*	Finland
31	MZ623074	GP.98382	*Bethylusfuscicornis*	Finland
32	MZ625448	GP.98383	*Bethylusfuscicornis*	Finland
33	MZ625629	GP.98386	*Bethylusfuscicornis*	Finland
34	MZ628620	GP.98390	*Bethylusfuscicornis*	Finland
35	MZ625827	GP.98392	*Bethylusfuscicornis*	Finland
36	MZ628568	GP.98393	*Bethylusfuscicornis*	Finland
37	MZ624001	GP.98395	*Bethylusfuscicornis*	Finland
38	MZ628150	GP.98396	*Bethylusfuscicornis*	Finland
39	MZ626974	GP.98398	*Bethylusfuscicornis*	Finland
40	MZ622996	GP.98401	*Bethylusfuscicornis*	Finland
41	MZ622772	GP.98403	*Bethylusfuscicornis*	Finland
42	MZ628250	GP.98404	*Bethylusfuscicornis*	Finland
43	MZ622905	GP.98407	*Bethylusfuscicornis*	Finland
44	MZ624628	GP.98409	*Bethylusfuscicornis*	Finland
45	MZ626853	GP.98410	*Bethylusfuscicornis*	Finland
46	MZ627512	GP.98411	*Bethylusfuscicornis*	Finland
47	MZ625571	GP.98412	*Bethylusfuscicornis*	Finland
48	MZ624577	GP.98414	*Bethylusfuscicornis*	Finland
49	MZ629043	GP.98418	*Bethylusfuscicornis*	Finland
50	MZ625429	GP.98424	*Bethylusfuscicornis*	Finland
51	MZ627966	GP.98427	*Bethylusfuscicornis*	Finland
52	MZ627958	GP.98431	*Bethylusfuscicornis*	Finland
53	MZ623543	GP.98432	*Bethylusfuscicornis*	Finland
54	MZ624158	GP.98436	*Bethylusfuscicornis*	Finland
55	MZ628700	GP.98437	*Bethylusfuscicornis*	Finland
56	MZ623872	GP.98438	*Bethylusfuscicornis*	Finland
57	MZ623157	GP.98441	*Bethylusfuscicornis*	Finland
58	MZ627785	GP.97434	*Goniozusclaripennis*	Finland
59	KF027190	NZAC04034054	*Goniozusjacintae*	New Zealand
60	KM216267	CUGN 01	*Goniozusnephantidis*	India
61	MN475311	1316	*Goniozusomanensis*	Oman
